# Establishment of a One Health workforce for pandemic prevention, preparedness and response

**DOI:** 10.2471/BLT.25.294954

**Published:** 2026-08-26

**Authors:** Ong-orn Prasarnphanich, Carla Berger, Philip AbdelMalik, Uzma Bashir, Raquel Medialdea Carrera, Renee Christensen, Sebastien Cognat, Virginie Dolmazon, Stacie Dunkle, Siobhan Fitzpatrick, Melinda Frost, Paula Gomez, Stefany Ildefonso, Gunel Ismayilova, Sylvio Menna, Marion Muehlen, Pryanka Relan, Flavio Salio, Chadia Wannous, Mathieu Boniol, Stéphane de la Rocque

**Affiliations:** a Health Security Preparedness Department, World Health Organization, Avenue Appia 20, 1211 Geneva 27, Switzerland.; b World Health Organization Hub for Pandemic and Epidemic Intelligence, Berlin, Germany.; c Health Emergency Alert and Response Operations Department, World Health Organization, Geneva, Switzerland.; d Country Readiness Strengthening Department, World Health Organization, Lyon, France.; e Surveillance System Department, World Health Organization, Geneva, Switzerland.; f Health Workforce Department, World Health Organization, Geneva, Switzerland.; g Animal Production and Health Division, Food and Agriculture Organization of the United Nations, Rome, Italy.; h One Health and Global Frameworks Department, World Organisation for Animal Health, Paris, France.

Countries are working to strengthen their health systems to enhance pandemic prevention, preparedness, response and resilience as they recover from the impacts of the coronavirus disease 2019 (COVID-19) pandemic. Achieving these objectives requires a One Health approach integrating sectors beyond human health, particularly where pathogens are shared among humans, animals and the environment. Effective pandemic preparedness and response are supported by collaborative surveillance^1^ and an adaptable, well-trained health workforce. The *WHO pandemic agreement* adopted in May 2025 calls for strengthening the workforce at the human-animal-environment interface and fostering a multidisciplinary, skilled global health and care workforce.^2^

In this article, we highlight how essential public health functions enable cross-sectoral collaboration^3^ and we underscore the importance of systematic workforce development. We also introduce the *Workforce development for effective management of zoonotic diseases: an operational tool of the tripartite zoonoses guide*,^4^ developed to support countries to strengthen a One Health workforce, in alignment with other established frameworks. 

To guide decision-making and improve health system resilience, in 2022, the World Health Organization (WHO) proposed 12 globally relevant essential public health functions.^3^ The workforce responsible for delivering these functions includes core public health personnel, health and care workers, and professionals from related sectors such as animal health, environmental science and the social sciences. The 12 functions are: (i) public health surveillance and monitoring; (ii) public health emergency management; (iii) public health stewardship; (iv) multisectoral planning, financing and management for public health; (v) health protection; (vi) disease prevention and early detection; (vii) health promotion; (viii) community engagement and social participation; (ix) public health workforce development; (x) health service quality and equity; (xi) public health research, evaluation and knowledge; and (xii) access to and utilization of health products, supplies, equipment and technologies.

While national workforce development strategies and training programmes are often sector-specific, effective pandemic response requires collaboration among relevant authorities and experts. A balanced, multisectoral workforce approach helps address risks at the animal–human–environment interface, thus reducing the likelihood of events with pandemic potential.

In the same roadmap, WHO developed a core approach for scoping, defining and strengthening the capacity of the public health and emergency workforce. This approach comprises three key components. First, defining expected functions and services. Second, strengthening competency-based education aligned with job responsibilities. Finally, estimating workforce supply and demand through mapping and measuring relevant occupations.^3^

Building on this foundation, the Food and Agriculture Organization of the United Nations (FAO), WHO and the World Organisation for Animal Health (WOAH) adapted WHO’s core approach and collaborated to develop an operational tool of the tripartite zoonoses guide.^4^ The tool supports countries in reviewing the competencies required to perform essential functions within existing mandates, frameworks and occupational roles.

The tool also identifies occupations across sectors involved in zoonotic disease management and includes a database of competency-based training options, with an emphasis on tripartite (FAO, WHO and WOAH) training, to support effective function delivery. Additionally, the tool provides additional resources to help countries address workforce-related challenges such as governance, labour market dynamics and occupational regulations.

Importantly, the tool aligns with WHO’s broader roadmap for developing national workforces capable of delivering the essential public health functions. The tool is complemented by a suite of topic-specific workforce development initiatives and competency frameworks, each with distinct objectives and target audiences. These frameworks include the *Global competency and outcomes framework for the essential public health functions*;^5^ the *Competencies for One Health field epidemiology (COHFE) framework*;^6^ the *GOARN competency model: for team member and team leader deployees*;^7^ the *Laboratory leadership competency framework*;^8^ the *Rapid response teams competency framework;*^9^ and the *Public health intelligence competency fram*e*work*.^10^

Authors from multiple WHO teams, responsible for developing and implementing competency frameworks that support a multisectoral, One Health approach, convened to explore presenting an overview of these frameworks to strengthen health-related workforce development. Each team contributed concise descriptions of the frameworks and related initiatives with additional details synthesized in Table 1. Subsequently, focal points from FAO and WOAH were invited to review and contribute.

**Table 1:** Summary of competency frameworks supporting multisectoral workforce development for pandemic prevention, preparedness and response

Title of competency framework, year	Description	Objective(s)	Target users	Learning audience	Countries where it has been piloted or implemented as of 15 January 2026
Workforce development for effective management of zoonotic diseases: an operational tool for the tripartite zoonoses guide,^4^ 2024	The framework provides sets of technical and nontechnical competencies required to manage zoonotic diseases effectively	To define competencies required by a multisectoral workforce for effective zoonotic disease management using a One Health approach	Government agencies with functions relevant to zoonotic disease management at national and subnational levels	Government personnel with functions relevant to zoonotic disease management at national and subnational levels	Country level: Albania, Côte d'Ivoire, Ethiopia, Jordan, Kazakhstan, Rwanda, Tajikistan Subregional level: Training-of-trainers for West Africa (Benin, Cabo Verde, Côte d’Ivoire, Gambia, Ghana, Guinea, Guinea Bissau, Liberia, Nigeria, Senegal, Sierra Leone, Togo); Training-of-trainers for Southern and Eastern Africa (Botswana, Ethiopia, Kenya, Malawi, Mozambique, Namibia, Rwanda, Somalia, Uganda, United Republic of Tanzania, Zambia, Zimbabwe)
Global competency and outcomes framework for the essential public health functions,^5^ 2024	The framework outlines competencies and practice activities for the public health and emergency workforce and provides curriculum guides and guidance on competency-based education	To provide a foundational tool and guide the strengthening of competency-based education approaches for core public health personnel, health and care workers, and occupations allied to health. To define the competencies and practice activities required to deliver the 12 globally defined essential public health functions, encompassing the whole of the public health workforce To guide the alignment of education programmes with employment towards the delivery of the essential public health functions	Educators and education institutions providing training of the public health workforce Employers and regulators of the public health workforce Anyone with an interest in the education of the public health workforce, including learners and practitioners themselves	Individuals who contribute to the delivery of at least one of the essential public health functions as part of integrated services and systems including: – Core public health personnel – Health and care workers – Occupations allied to health	Not country specific
Competencies for One Health field epidemiology (COHFE) framework,^6^ 2023	Developed jointly by WHO, FAO and WOAH, the framework defines One Health competencies for field epidemiologists across the public health, animal health and environment sectors	To define the knowledge, skills and competencies needed for field epidemiologists to implement the One Health approach To describe the cross-cutting One Health and sector-specific knowledge, skills and competencies To provide guidance for One Health field epidemiology curriculum development, mentorship, learning evaluation and certification, and continuing education programmes	Field epidemiology training programme directors, resident advisors and staff Workforce development units across human and animal health and environment sectors One Health policy-makers International and regional partner organizations	Field epidemiology training programme participants National and subnational public health, animal health and environment sector professionals	Country level: Bangladesh, India, Nigeria
GOARN competency model: for team members and team leader deployees,^7^ 2017	The framework defines necessary skills and behavioural indicators required of responders serving as surge capacity. This WHO surge capacity network comprises over 310 technical institutions and networks globally that respond to acute public health events with the deployment of staff and resources to affected countries	To define the necessary core cross-functional or soft skills required of individuals serving in surge capacity to national and international public health emergency responses To equip the surge workforce to work effectively and efficiently within and across sectors and levels of a response	Organizations and entities involved in public health emergency workforce development Organizations and entities involved in national and international surge capacity	Representatives of the network’s partner institutions working in national and international public health emergency response: governmental and NGOs, including national public health institutes, UN agencies, academic institutions and other relevant agencies	Not country specific
Laboratory leadership competency framework,^8^ 2019	The framework outlines competencies for laboratory leaders working across human, animal and environmental health laboratories	To outline the essential competencies needed by laboratory leaders to build, strengthen and direct sustainable national laboratory systems for disease detection, control and prevention in health systems	National or regional authorities from all health sectors and disciplines Regulatory agencies Educational institutions NGOs and private sector organizations contributing to the laboratory system	Current and emerging laboratory leaders working in human, animal and environmental health laboratories	Country level: Armenia, Burkina Faso, Chad, Ecuador, Ghana, Guinea, Kazakhstan, Kyrgyzstan, Liberia, Malawi, Mali, Republic of Moldova, Mozambique, Oman, Pakistan, Paraguay, Sierra Leone, Tajikistan, Uganda, United Republic of Tanzania, Ukraine, Uzbekistan, Viet Nam, Zambia Subregional level: Central Africa (Central African Republic, Chad, Democratic Republic of the Congo, Gabon, Republic of the Congo); South-East Asia (Cambodia, Indonesia, Malaysia, Thailand)
Rapid Response Teams competency framework,^9^ 2024	The framework outlines areas of operation and competencies for rapid response teams during preparedness and response phases	To outline essential rapid response team areas of operation during preparedness and response phases To provide a common understanding of knowledge, skills and attitudes required by individual rapid response team members, managers and trainers To define competencies required for operational success	Educators and institutions providing education and training of the emergency workforce Employers of the emergency workforce	Health ministries National disaster management agencies Emergency operations centres and public health emergency operations centres Incident management teams, etc. National public health agencies Local academic institutions NGOs and partners National professionals (within and outside the health sector) who are likely to be deployed as rapid response team members or serve as managers and trainers	Country or territory level: Afghanistan, Algeria, Angola, Benin, Botswana, Burundi, Central African Republic, Comoros, Congo, Djibouti, Egypt, Equatorial Guinea, Eswatini, Ethiopia, Iran (Islamic Republic of), Iraq, Jordan, Kuwait, Lebanon, Lesotho, Libya, Madagascar, Mauritius, Morocco, Mozambique, Namibia, Nepal, Nigeria, Oman, Pakistan, Rwanda, Sao Tome and Principe, Saudi Arabia, Somalia, South Sudan, Sudan, Syrian Arab Republic, Togo, Tunisia, Uganda, United Arab Emirates, West Bank, Yemen, Zambia
Public health intelligence competency framework,^10^ 2025	The framework provides standardized competencies for conducting public health intelligence using an all-hazards, One Health approach	To strengthen capacity for a unified all-hazards, One Health approach to early detect, verify, assess and communicate public health threats To develop and maintain standardized competencies required by the workforce in countries, WHO, NGOs and networks worldwide to conduct public health intelligence	Educators Institutions providing public health training Governmental organizations NGOs Policy-makers Licensing and regulatory agencies Academic institutions International and regional partner organizations	Professionals involved in public health intelligence activities at all levels Employers and supervisors involved in hiring, evaluation and workforce planning Policy-makers and public health leaders Training institutions and educators (universities, field epidemiology training programmes, public health schools) Learners: new, mid-career or advanced public health practitioners Organizations involved in surveillance, emergency response, One Health, and multisectoral intelligence	Country level: Armenia, Djibouti, Egypt, Jordan, Lebanon

FAO: Food and Agriculture Organization; GOARN: Global Outbreak Alert and Response Network; NGO: nongovernmental organization; UN: United Nations; WHO: World Health Organization; WOAH: World Organisation for Animal Health.

As WHO engages with Member States, the competency frameworks supporting a One Health approach presented here are implemented primarily at the national level, with flexibility for use at subnational levels. Most of these frameworks (including the competency and outcomes frameworks) encompass both technical and nontechnical content. In contrast, the Global Outbreak Alert and Response Network model^7^ focuses exclusively on nontechnical content. Technical content is specific to functions or thematic areas such as surveillance, risk assessment and data analysis. Nontechnical content, such as decision-making, communication, leadership and management, is cross-cutting and applicable to personnel working across diverse domains.

The One Health field epidemiology framework articulates competencies across frontline, intermediate and advanced levels and maps them as cross-cutting One Health competencies or as specific to the public health, animal health or environment sector.^6^ Other competency frameworks adopt a more generalized approach, without explicit differentiation by application level or sector. To support multisectoral workforce development, such general frameworks can be applied alongside sector-specific instruments, including WOAH’s roadmap for veterinary workforce development and the *Competency and curriculum guidelines for community animal health workers*.^11^ This complementary use of frameworks underscores the principle that effective governance of One Health workforce development depends on shared responsibility and coordinated action across sectors.

Member States may draw on different competency frameworks to strengthen workforce capacity in line with their specific needs and priorities. Several initiatives^12^^–^^14^ already offer online and in-person training programmes aligned with defined competency areas, enabling countries or organizations to readily nominate or assign personnel who would benefit from such training. Although the operational tool of the tripartite zoonoses guide does not provide training directly, its supplemental database serves as a repository of training options and tools to support the development of corresponding competencies. In addition, the frameworks for essential public health functions, competencies for One Health field epidemiology and public health intelligence competency, as well as the global laboratory leadership programme provide guidance on curriculum development linked to their respective frameworks. As the competency and outcomes framework for essential public health functions presents the breadth of content applicable across a broad range of health and sectors allied to health, countries may apply this framework flexibly and design workforce strengthening initiatives using approaches that best suit their national contexts.

Based on our experience, some health ministries, using a multisectoral approach, begin by conducting multisectoral reviews of workforce development needs and gaps, which then inform the application of competency frameworks and the development of workforce strengthening plans. Planning and actions grounded in a multisectoral assessment of the workforce are essential to avoid duplicative use of frameworks and fragmented, sector-specific implementation. An overarching workforce analysis and strategic planning may be followed by more targeted focus on specific technical areas. Accordingly, countries need only apply those frameworks that are relevant to their identified needs or select from the relevant components of the different frameworks. In addition, some countries may have established national frameworks and therefore choose to review and complement them using the frameworks presented here.

For example, after reviewing existing or previous national workforce assessments and strategies, Côte d’Ivoire and Jordan applied the operational tool of the tripartite zoonoses guide to analyse their One Health workforce, identifying epidemiology and joint risk assessment, respectively, as priority areas for capacity-building;^15^ relevant guidance from the *Competencies for One Health field epidemiology (COHFE) framework,*^6^ and training options listed in the tool’s database could potentially be leveraged to address relevant competencies. The ministries involved in establishing or strengthening the One Health workforce should establish a monitoring and evaluation mechanism to track progress and identify opportunities to strengthen programme implementation. Furthermore, workforce strategies ideally incorporate incentivization, personnel’s recognition and retention measures to ensure that workforce capacity is both developed and sustained.

No single approach for applying these frameworks exists, as they are intended to provide common sets of competencies relevant to diverse contexts, priorities and occupational groups. Beyond direct workforce training, the frameworks can be used to assess existing capacity-building initiatives, identify areas for improvement and support alignment with international standards.

WHO’s workforce development approach is designed to be adaptive and progressive rather than prescriptive, reflecting the diversity and complexity of multisectoral, One Health workforce realities across countries. By drawing attention to a range of existing frameworks that may inform workforce development, we seek to encourage reflection and strategic deliberation, supporting countries as they navigate choices and align workforce development planning with nationally defined priorities and constraints.
